# Supplementary material for: Care When It Counts: Establishing Trauma-Sensitive Care as a Preventative Approach for 0–3-Year-Old Children Suffering from Trauma and Chronic Stress
Source: Children (Basel). 2023 Jun 8;10(6):1035. doi: 10.3390/children10061035 (PMC10297293; doi:10.3390/children10061035)
Supplement: Supplementary file 1 [file children-10-01035-s001.zip › children-2348372-supplementary.pdf]

Table S1. Quantitative questionnaire

Please, answer the following questions as sincerely as possible. Express your agreement with each of the following sentences by using the following scale:

1	2	3	4	5
Completely disagree	***Disagree***	***Neutral***	***Agree***	***Completely agree***

		Strongly disagree (1)	Disagree (2)	Neutral (3)	Agree (4)	Strongly agree (5)
1	Trauma symptoms are visible in babies and toddlers.					
2	Trauma is not only related to physical and sexual abuse but also to psychological maltreatment and neglect.					
3	Domestic violence during infancy can have long-term effects on physical, emotional and mental health later in life.					
4	I have enough knowledge to detect signs of trauma in young children.					
5	I have enough knowledge to understand the effects of trauma on infants.					
6	I am aware of the referral procedures to follow in case of suspected abuse.					
7	I have a comprehensive understanding of Trauma Sensitive Care.					
8	Childcare professionals can play an important role in screening and referring child abuse.					
9	I believe in and support the principles of Trauma Sensitive Care.					
10	I share my expertise and collaborate effectively with colleagues regarding the use of Trauma Sensitive Care.					
11	I would like to receive more training on Trauma Sensitive Care.					
12	I feel confident I have the skills to regulate an infant's stress response.					
13	I feel confident I have the skills to restore the connection with an infant.					
14	I feel confident I have the skills to provide reasoning and help the child gain insight after a stressful event.					
15	I know strategies to engage and soothe children during moments of crisis.					
16	I feel confident I have the skills to regulate a primary caregiver's stress response.					

17	I feel confident I have the skills to restore connection with a primary caregiver.					
18	I feel confident I have the skills to provide reasoning and help the primary caregiver gain insight after a stressful event.					
19	I feel equipped to communicate with the primary caregiver after detecting signs of domestic violence.					
20	I feel equipped to refer the family to specialized organizations after detecting signs of domestic violence.					
21	I feel confident I have the skills to regulate my own stress response when dealing with a stressful situation with a child or primary caregiver.					
22	I practice self-care (taking care of my own needs and well-being).					
23	I feel I can share my concerns about possible signs of domestic violence with my colleagues.					
24	I experience trust and safety in my current work environment.					
25	I found the Trauma Informed Care Protocol to be useful.					
26	I found the Trauma Informed Care Protocol to be easy to comprehend.					
27	I intend to use the Trauma Informed Care Protocol.					
28	The content of the program aligned with my expectations.					
29	I am satisfied with the content of the program.					
30	I am satisfied with the way of teaching.					
31	I am satisfied with the learning material.					
